# Analysis of the Chemical Distribution of Self-Assembled Microdomains with the Selective Localization of Amine-Functionalized Graphene Nanoplatelets by Optical Photothermal Infrared Microspectroscopy

**DOI:** 10.1021/acs.analchem.2c02306

**Published:** 2022-08-16

**Authors:** Suihua He, Pascaline Bouzy, Nicholas Stone, Carwyn Ward, Ian Hamerton

**Affiliations:** † Bristol Composites Institute, Department of Aerospace Engineering, School of Civil, Aerospace, and Mechanical, Engineering, University of Bristol, Queen’s Building, University Walk, Bristol BS8 1TR, U.K.; ‡ Physics and Astronomy, College of Engineering, Mathematics and Physical Sciences, University of Exeter, Exeter EX4 4QL, U.K.

## Abstract

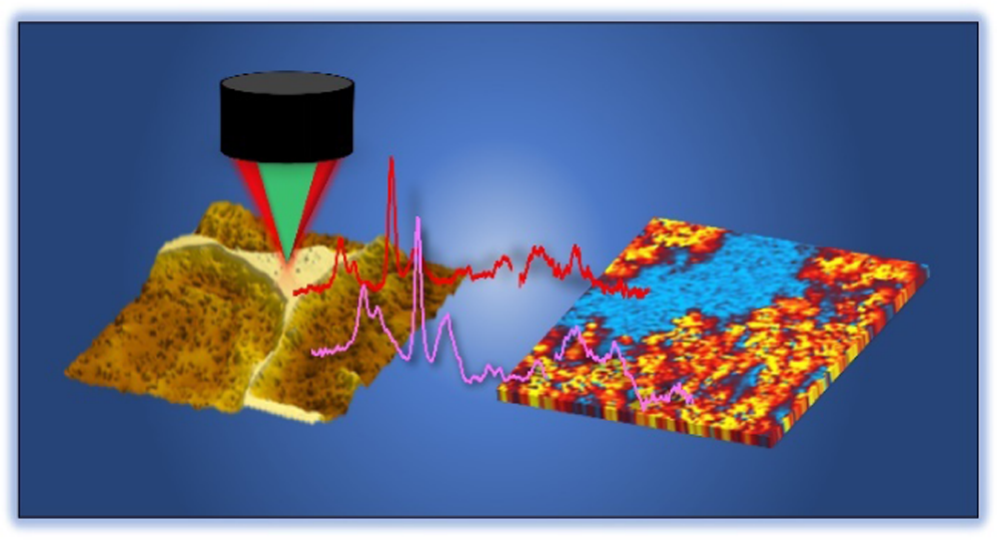

By incorporating 1-(2-aminoethyl)piperazine (AEPIP) into a commercial epoxy blend, a bicontinuous microstructure is produced with the selective localization of amine-functionalized graphene nanoplatelets (A-GNPs). This cured blend underwent self-assembly, and the morphology and topology were observed *via* spectral imaging techniques. As the selective localization of nanofillers in thermoset blends is rarely achieved, and the mechanism remains largely unknown, the optical photothermal infrared (O-PTIR) spectroscopy technique was employed to identify the compositions of microdomains. The A-GNP tends to be located in the region containing higher concentrations of both secondary amine and secondary alcohol; additionally, the phase morphology was found to be influenced by the amine concentration. With the addition of AEPIP, the size of the graphene domains becomes smaller and secondary phase separation is detected within the graphene domain evidenced by the chemical contrast shown in the high-resolution chemical map. The corresponding chemical mapping clearly shows that this phenomenon was mainly induced by the chemical contrast in related regions. The findings reported here provide new insight into a complicated, self-assembled nanofiller domain formed in a multicomponent epoxy blend, demonstrating the potential of O-PTIR as a powerful and useful approach for assessing the mechanism of selectively locating nanofillers in the phase structure of complex thermoset systems.

## Introduction

Epoxy-based thermosetting resins have found wide use throughout a variety of automotive, aerospace, and military applications due to their ease of processing, good dimensional stability, and excellent electrical and chemical resistance.^1−4^ Incorporating nanofillers into polymer matrices remains the most effective and popular approach for the fabrication of multifunctional epoxy composites, which has been attributed to the possibility of mass production and easy processing.^5,6^ However, performance improvements are limited due to the randomly distributed state of the nanofillers, whereas the formation of a highly efficient filler network is very important for the structural design of multifunctional composites. Selective localization of nanofillers in the continuous phase in the phase-separated structure has proven to be one of the most effective and promising methods to fabricate materials with desired properties.^7−10^

In recent decades, reinforcing epoxy resins by the introduction of engineering thermoplastics has stimulated much research because of enhancing toughness without sacrificing thermal stability or reducing glass-transition temperature (*T*_g_) significantly. Typically, engineering thermoplastics, such as polyethersulfone,^11,12^ polysulfone,^13^ and polyetherimide (PEI),^14,15^ display good compatibility with the epoxy oligomers but undergo gradual separation from the epoxy matrix during the polymerization to form a second phase. This so-called polymerization-induced phase separation (PIPS) offers a pathway for generating thermoset polymers with well-defined nanomicrostructures *via* the spontaneous segregation of otherwise miscible components upon an increase in the molecular weight of at least one of the components during polymerization.^16−18^ For instance, Wang et al.^9^ reported that the selective localization of multiwall carbon nanotubes (MWCNTs) in the cocontinuous phase structure in an epoxy/PEI system was able to improve the mechanical, thermal, and electrical properties, simultaneously. Among the increased comprehensive performance, the most significant enhancement is that the volume resistivity decreased from 3.29 × 10^15^ to 3.86 × 10^6^ Ω·m at 2.0 wt % MWCNTs due to the formation of a self-assembled filler network *via* PIPS.

However, an inevitable problem with these materials is the poor interface and adhesion between the two different phases, which can lead to a reduction in the strength of the interface. Most importantly, the processability and thermostability of thermoset resins deteriorate due to the presence of the high molecular weight of thermoplastics. Hence, the exploration of selectively locating nanofiller in thermoset (TS)/thermoset (TS) systems is expected for unlocking these materials’ potential applications. Nevertheless, when compared to systematically studied thermoplastic (TP)/TS systems, the selective localization of nanofiller in TS/TS blend was achieved and investigated very recently by Huang and co-workers.^19^ They reported that the MWCNTs selectively located in the continuous domain dramatically improved the toughness of the epoxy resin and the electrical properties of epoxy composites containing MWCNTs while maintaining excellent tensile strength and modulus. To point out the mechanism of the selective localization of MWCNTs in the TS/TS blend, the interfacial energy between MWCNTs and two different types of epoxy resin, the diglycidyl ether of bisphenol A (DGEBA) and tung oil-based diglycidyl ester (TODGE), was measured. A lower interfacial energy between MWCNTs and DGEBA was observed, which normally implies a stronger affinity between them.

Recently, strategies to achieve phase structure in TS blends have been investigated by several researchers to ascertain the factors influencing the phase separation and the morphology–structure relationships in thermoset blends. Wang et al.^20^ fabricated benzoxazine/bismaleimide blends with bicontinuous phase structures *via* an imidazole catalyst and found that both the phase separation and phase morphology were mainly determined by the viscosity of blends. Yue et al.^21^ investigated the PIPS behavior in benzoxazine/epoxy systems and the results showed that it was possible to effect the sequential polymerization of the epoxy resin and the benzoxazine either by increasing the initial molecular weight of the epoxy component or by adding imidazole as the catalyst, resulting in a phase-separated structure. Despite much progress in understanding the structure, properties, and phase behavior of TS/TS blends, these studies relied heavily on imaging and thermal analysis techniques, while direct evidence about the distribution of each component at the molecular level was lacking.

Optical photothermal infrared (O-PTIR) spectroscopy is an analytical technique that is still emerging in recent years and can provide chemical images in a relatively fast manner with submicron spatial resolution (order of magnitude below the diffraction limit of IR frequencies measured). This powerful technique with the working principle based on measuring the photothermal response of a sample illuminated by infrared radiation,^22,23^ operating in a noncontact microscopy mode and offering significantly higher sensitivity and resolution than FTIR spectroscopy,^24^ opens a new avenue for the nondestructive, efficient, and reliable analysis on living cells and organisms.^25^ However, to the best of the authors’ knowledge, O-PTIR has been rarely employed in polymer science. Not to mention the fact that the chemical analysis of phase structure in TS/TS blends is much more complicated as the copolymerization between the two components tends not to favor the formation of a discrete second phase. Consequently, while it plays an essential role in understanding the mechanism of the PIPS in TS blends,^26,27^ it is rarely studied. In the present work, we report the use of O-PTIR spectroscopy to identify the chemical distribution of a self-assembled phase domain with the selective localization of amine-functionalized graphene during the cure of a liquid-processable commercial epoxy blend. The possibility of using this powerful technique to investigate the plausible mechanisms of phase separation in TS systems is summarized, which might enable the controlling of the phase structure with the selective localization of nanofillers in TS blends.

## Experimental Section

### Materials

**Figure 1:**
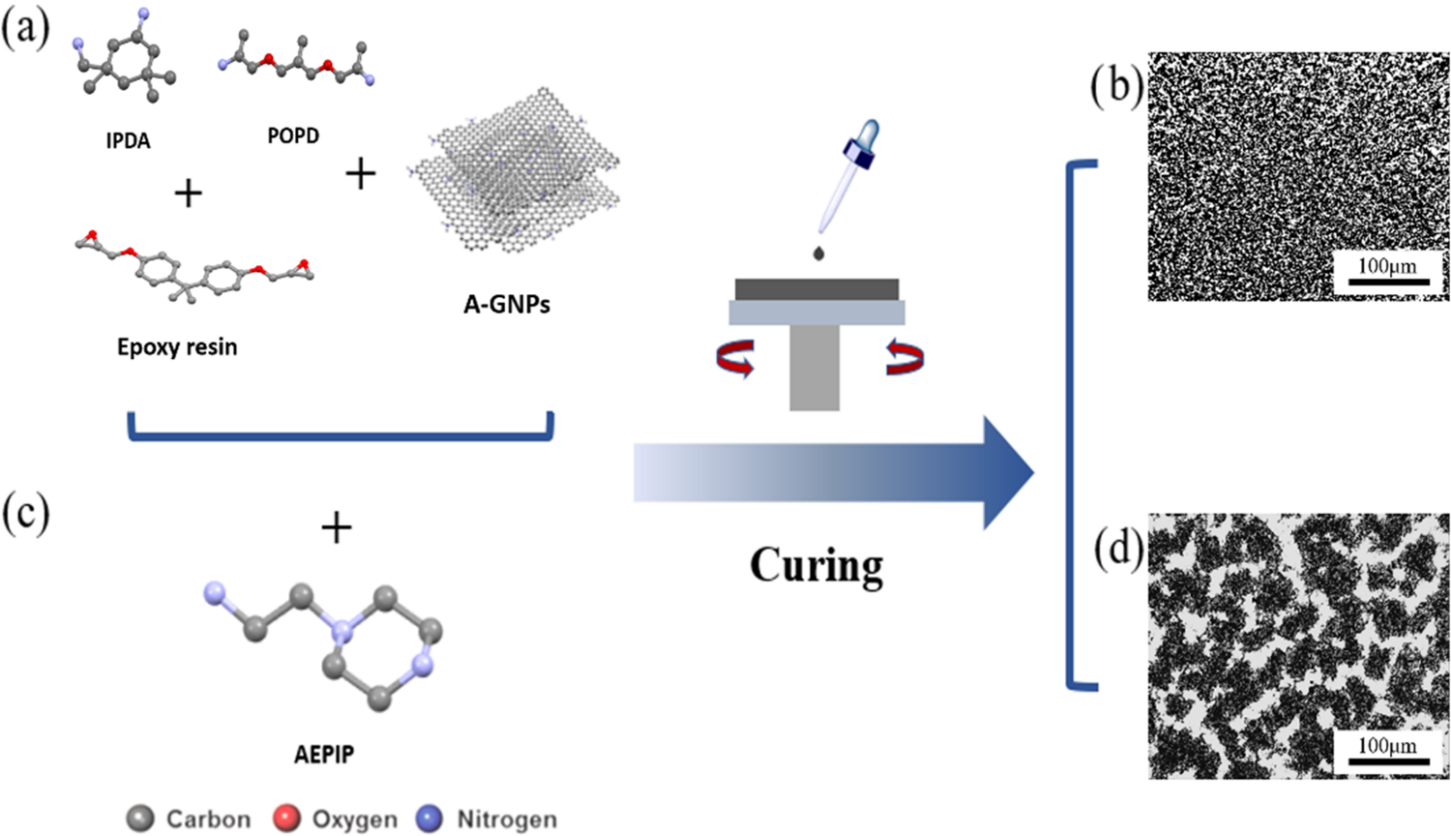
Fabrication process and structure of the phase-separated domain with A-GNPs, (a) A-GNPs dispersed in IPDA and POPD *via* ultrasonication and (c) AEPIP incorporated in the mixture before mixing with the epoxy blend for fabricating nanocomposite films. (b, d) Corresponding optical images of the films fabricated by the multicomponent blend.

Component A, RS-M135 (PRF Composites, U.K.), is an epoxy resin produced from bisphenol A diglycidyl ether (DGEBA) (CAS no. 25068-38-6) with a number average molecular weight, Mn < 700 g/mol. (70–90% w/w) and containing an added proportion of 1,6-hexanediol diglycidyl ether (DGEH) (CAS no. 16096-31-4) as a reactive diluent. Component B, RS-MH137 (PRF Composites, U.K.), which is a hardener, contains (a) isophorodiamine abbreviated as IPDA (3-aminomethyl-3,5,5-trimethylcyclohexylamine) (CAS no. 2855-13-2) 35–50% w/w and (b) poly(oxypropylenediamine) abbreviated as POPD (CAS no. 9046-10-0) 50–70% w/w. Component C, 1-(2-aminoethyl)piperazine (AEPIP), was purchased from Sigma-Aldrich (CAS no. 140-31-8). The chemical structure of the main components used appears in Figure 1. Amine-functionalized, few-layer graphene nanoplatelets (A-GNPs) with a mean diameter of 2 μm and a thickness under 4 nm were purchased from Cheap Tubes Inc. and used as reinforcement in this study. According to the manufacturer, these graphene nanoplatelets were produced by the mechanical exfoliation process and then surface-modified with >7% primary amino (NH_2_) functional groups. All of the materials in this study were used as received without further purification.

### Sample Preparation

To process the materials in the present study, first, graphene nanoplatelets (3 wt %) were dispersed in the curing agents in the appropriate ratio by sonication probe in a water bath at room temperature for 1 h. Before the mixing process, the neat epoxy was degassed *via* a vacuum line at 25 °C for 10 min. As shown in Figure 1, components A and B were mixed to fabricate a composite blend, named as A–B. Another set of samples was cured by the hardener with components B and C in 8:2 and 7:3 weight ratios, name as A–BC (8:2) and A–BC (8:3). The blends were then mixed using a mechanical stirrer for 10 min at 1000 rpm. Finally, the A-GNP/epoxy composite films were fabricated by repeated spin-coating followed by a postcure process. Briefly, the A-GNP/epoxy mixture was spin-coated on a glass substrate with a rotation speed of 1500 rpm. The composite films were kept at room temperature for 60 mins before a postcure process (60 °C for 4 h) was employed. The epoxy resin and hardener were mixed in a 10:3 weight ratio for all samples.

### Characterization

The distribution and dispersion of the GNPs in the epoxy matrix on a larger scale were studied using an optical transmission microscope (Zeiss Axio Imager 2, Carl Zeiss MicroImaging GmbH, Jena, Germany). Images were captured and then processed using ImageJ software (https://imagej.net/downloads). Scanning electron micrographs (TM3030Plus, Hitachi) were gathered under an acceleration voltage of 15 kV after samples were sputter-coated with gold. Atomic force microscopy (AFM) images were collected using a Dimension XR (Bruker, Santa Barbara) with an Icon scanner, operating in a peak force tapping mode (nominal spring constant of 0.4 N/m, peak resonant frequency of 2 kHz); only height images were recorded. AFM images were thresholded to get binary images, and the characteristics of different domains were evaluated. AFM images are processed by Gwyddion (version 2.59, http://gwyddion.net/), and the three-dimensional (3D) topology images, and a virtual detail of cross section is generated accordingly. Transmission electron micrographs (TEMs) were obtained on a Tecnai T12 (Thermo-Fisher) electron microscope at an accelerating voltage of 120 kV. The transverse sections of samples for electron microscopy were cut *via* an Ultracut E ultramicrotome. Sections were 80 nm thick and supported on grids coated with a pioloform film.

Bulk infrared spectra were acquired using a PerkinElmer Spectrum 100 FTIR spectrometer (Beaconsfield, U.K.). The spectrum range was 4000–600 cm^–1^, and 16 scans were acquired and coadded for each measurement. Spectrum software was used for the collection and analysis of IR spectra.

The newly emerging optical photothermal infrared spectroscopy (O-PTIR) technique was employed to distinguish each phase from one another and thus to further understand the chemical distribution in the phase-separated domain in such ternary films. O-PTIR measurements were carried out using a mIRage infrared microscope (Photothermal Spectroscopy Corp., Santa Barbara) equipped with a 40× objective (N.A. 0.78), a 4-module-pulsed QCL with a tunable range from 1799 to 785 cm^–1^, and the photothermal expansion was detected by indirectly measuring the associated change in refractive index by measuring the change in the scattering of a diode laser beam at a 785 nm wavelength.

In this work, single-point spectral data were acquired over the spectral regions 1799 and 785 cm^–1^ with a 6.6 cm^–1^ spectral resolution and 9 scans per spectrum. Single IR frequency images were collected in a reflective mode at a 500 nm step size by tuning the QCL device to the frequencies corresponding to the wavelengths of 1512, 1320, 1108, and 1030 cm^–1^; ratio images were created from the individual scans. Instrument control and data collection were performed using PTIR Studio 4.3 software supplied by the manufacturers.

## Results and Discussion

The schematic shown in Figure 1 depicts a facile procedure and representative chemical structures for fabricating the phase-separated structures from a previously homogeneous dispersed state. The combination of chemicals shown in Figure 1a is liquid-processable, the infusible commercial formulation used for the manufacture of wind turbine blades. In this particular case, A-GNPs are incorporated as reinforced particles. The A-GNPs are homogeneously dispersed in the epoxy matrix (Figure 1b), but after the incorporation of AEPIP (Figure 1c) into the chemical formulation, a continuous phase-separated domain with graphene nanoplatelets is self-assembled with a width in the range of 10–40 μm (Figure 1d).

**Figure 2:**
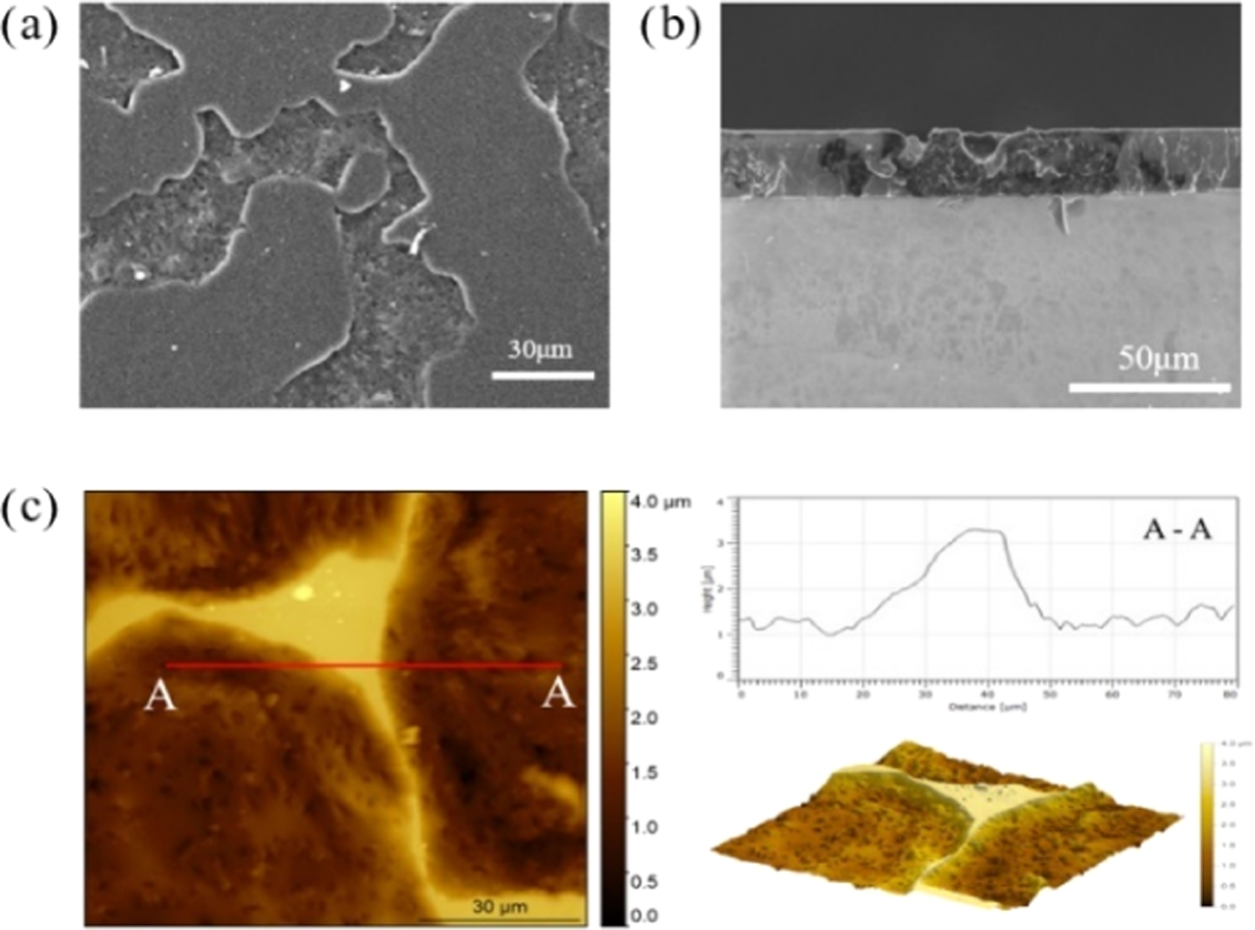
Corresponding SEM images of (a) top view, (b) cross section, and (c) AFM images of the final optically reconstructed morphology.

Scanning electron microscope (SEM) and AFM are employed to visualize the morphology and topology of the phase structure of the nanocomposite. Figure 2a,b presents the SEM images of the phase morphology of the different domains, which shows an obvious contrast between each domain, with one of them exhibiting a smooth surface and the other domain with a rippled surface. In addition, from the cross-sectional image, a composite film with a thickness of around 20 μm could be observed. To visualize the topology of this unexpected phase structure in detail, AFM characterization is applied for further exploration. AFM images (Figure 2c) demonstrate the topology of the phase-separated structure constructed in the epoxy nanocomposite film containing graphene nanoplatelets. This indicates that the domain with the smooth surface is epoxy-rich, while the domain with the rippled surface is associated with the presence of A-GNPs, which suggests that the nanoplatelets aggregate to form a dense domain during the curing process. Then, the aggregated nanosheets essentially drop into those channels to form a column, while the resin is deposited on either side of the graphene domain. Eventually, a 3D microstructure with surface roughness over 2 μm was constructed.

Owing to the complicated copolymerization between different components during the curing process, selective localization of nanofillers in phase-separated structures in TS/TS systems is rarely reported, the mechanism of which remains largely unknown. Thus, to investigate the chemical composition of these discrete phases of the multicomponent TS system, a novel noncontact imaging technique, the so-called O-PTIR spectroscopy, was employed to provide high sensitivity IR spectrum and access the chemical maps with a spatial resolution compositional distribution on a submicron scale. Prior to identifying the chemical dissimilarities of each domain by O-PTIR spectroscopy, bulk IR spectra were recorded *via* conventional FTIR spectroscopy to identify the chemical structure of each component.

**Figure 3:**
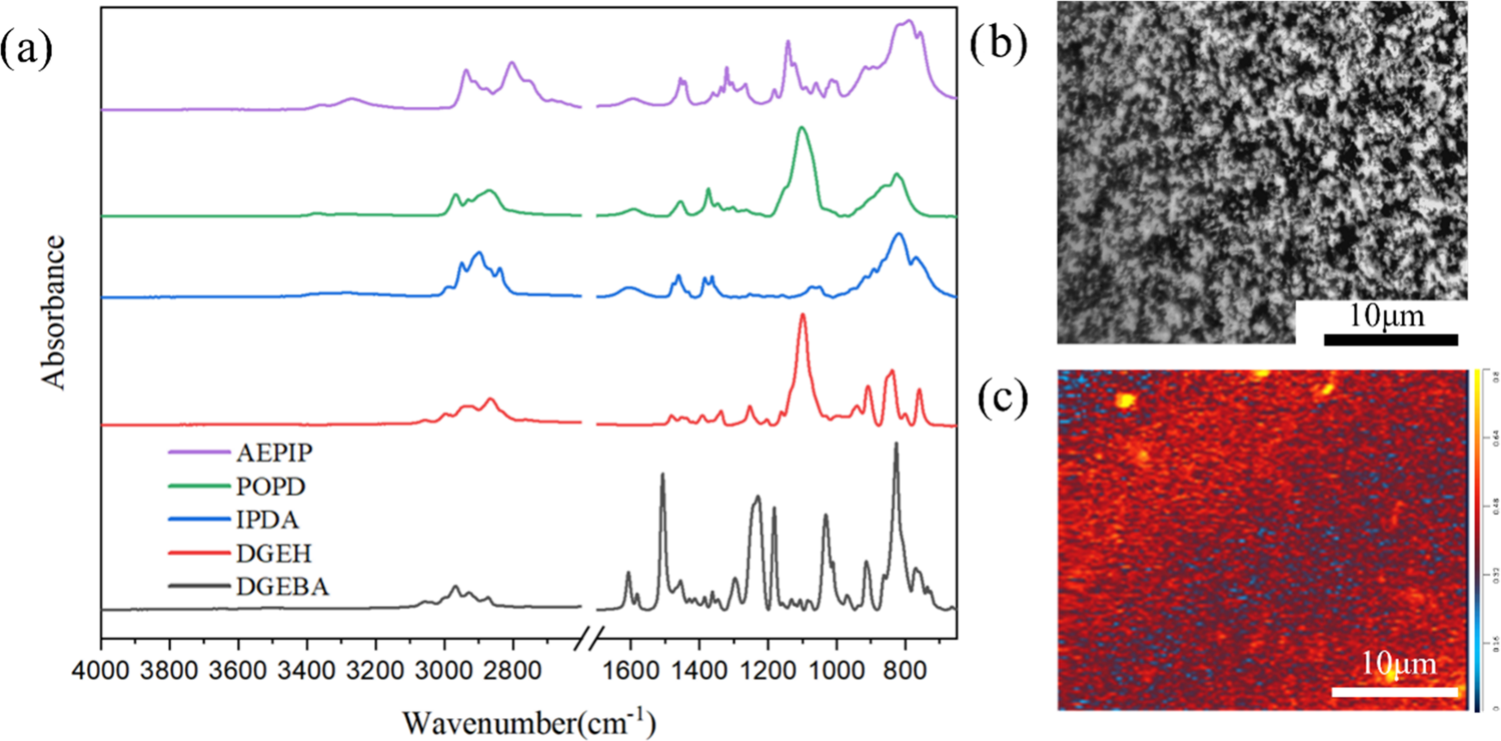
(a) FTIR spectrum of each component and (b) optical image and (c) chemical map (1030 cm^–1^/1512 cm^–1^) of the RM135/RS-MH137 blend with A-GNPs.

As seen in Figure 3a, even though there is no obvious IR peak for identifying the DGEH and curing agents, as the epoxy resin is mainly formed by the DGEBA, the distinct vibration of the aromatic ring in the 1512 cm^–1^ of DGEBA can be utilized as the spectral signature of the epoxy resin in these blends. Most importantly, the significant difference in the IR spectrum in the region of 1260–1320 cm^–1^, which is assigned to the vibration of the secondary or tertiary amine from the heterocyclic amines of AEPIP, could be used to identify the localization of AEPIP with the other two amine reagents from component B. The optical image and related chemical map of the RM135/RS-MH137 blend with A-GNP are shown in Figure 3b,c, respectively. In Figure 3b, it could be seen that the A-GNP is dispersed homogeneously in the epoxy matrix without any phase features, which is completely different from the phase-separated structure (Figure 2). Despite no obvious phase structure being seen in this system, for evaluating the chemical dissimilarity, a chemical map was constructed using the ratio of IR absorption images at 1108 cm^–1^ (C–H bending)/1512 cm^–1^. The IR peak at 1108 cm^–1^, which is assigned to the C–O bond of aliphatic ether, was selected to locate DGEH and POPD. Figure 3c depicts the corresponding chemical map, from which it was confirmed that no heterogeneous chemical distribution could be discerned at the microscale. For comparison, the corresponding IR spectrum of A-GNP is shown in Figure S1.

**Figure 4:**
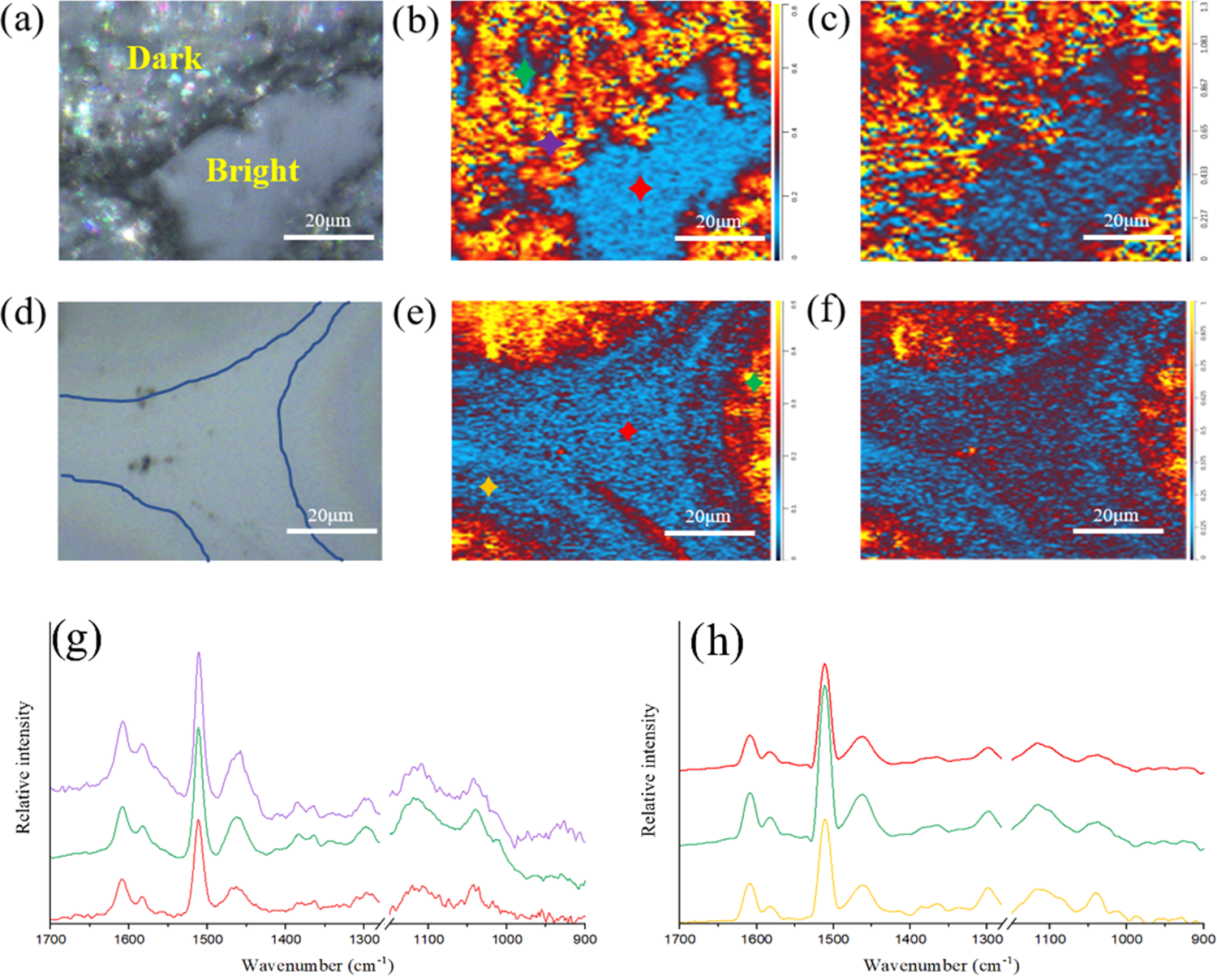
Optical image and chemical map obtained by constructing a ratio of IR absorption images at 1320 cm^–1^/1512 cm^–1^ and 1030 cm^–1^/1512 cm^–1^ of the A–BC (8:2) blend. (a–c) Incorporated with A-GNP and (d–f) without A-GNP. (g, h) O-PTIR absorption spectra obtained from different spots in panels (b, e) (colored stars represent the spots for acquiring IR spectra).

In this current work, as the A-GNPs were successfully located in the phase-separated microstructure from a uniformly dispersed state, it is believed that probing the chemical distribution *via* O-PTIR is the first and critical step before examining the underlying mechanism of this unexpected phase behavior, which is now underway. As shown in Figure 4a, the dark and bright domains are clearly shown and highlighted. It should be noted that when compared with the RM135/RS-MH137 blend, the blend containing AEPIP shows an obvious chemical contrast when the chemical map turns to the ratio of 1320 cm^–1^/1512 cm^–1^, which means that the graphene (dark) domain is displaying much stronger absorptions than the resin-rich (bright) domain at 1320 cm^–1^. In addition to the chemical map, the IR spectrum of some individual spots (highlighted in Figure 4b) was acquired. Comparing the O-PTIR spectra to FTIR reference spectra of each component (Figure 3a), it could be confirmed that the acquired O-PTIR spectra have very high quality and with less signal interference from the glass substrate (Figure 4g), which enables the precise detection of chemical differences in the submicron level. Even though the spectrum shows relatively similar intensities in the different domains, while the ratio constructed by different wavenumbers is clearly evidencing the chemical contrast between graphene-rich and epoxy-rich domains, the stronger signal of AEPIP is observed in the graphene domain.

**Figure 5:**
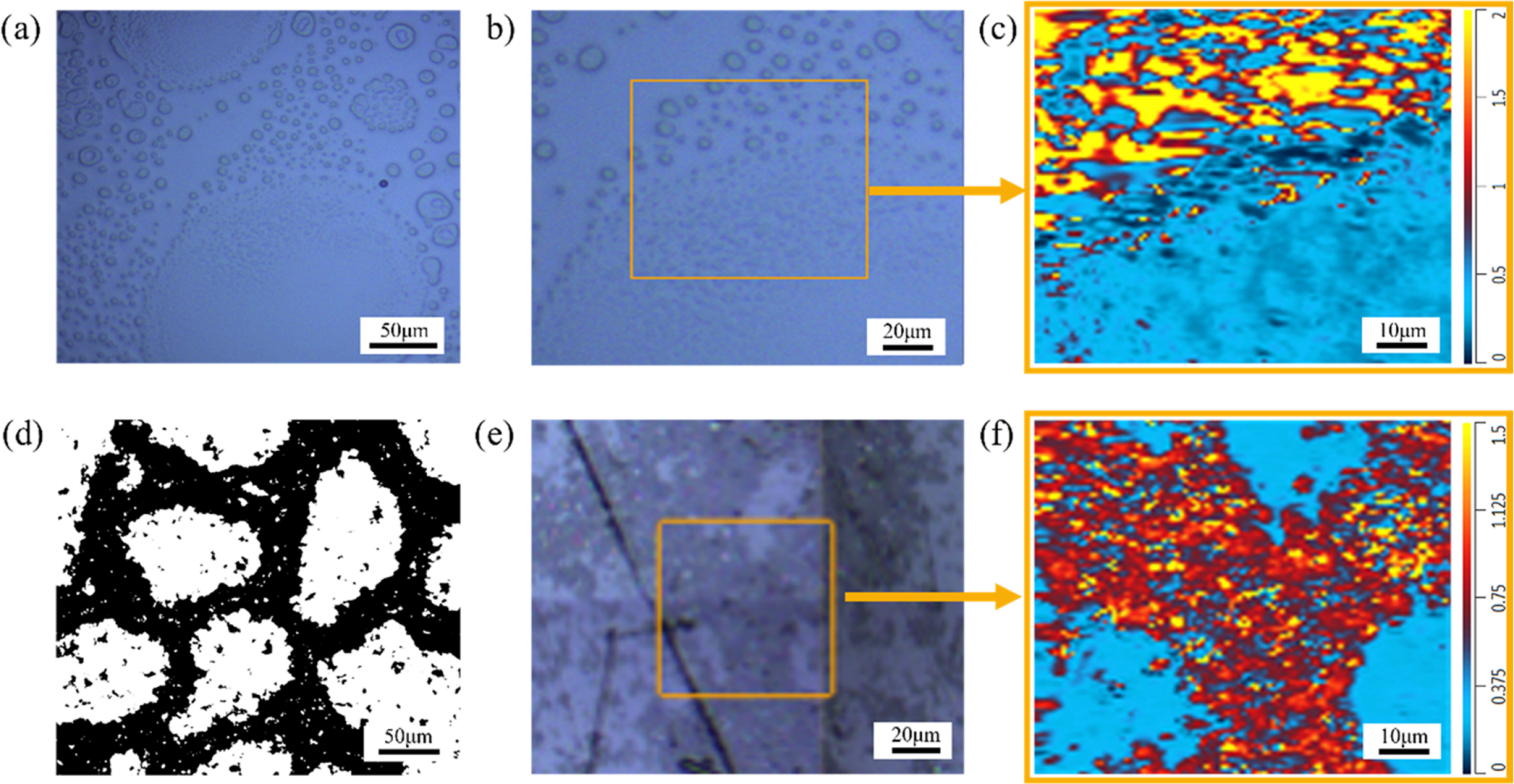
Optical image and chemical map (1320 cm^–1^/1512 cm^–1^) of the A–BC (7:3) blend (a–c) without A-GNP and (d–f) incorporated with A-GNP.

Similarly, Figure 4c illustrates the chemical map of 1030 cm^–1^/ 1512 cm^–1^; the wavenumber image was collected by tuning the QCL device to the frequencies of 1030 cm^–1^, which is assigned to the O–H vibration of secondary alcohol generated during the curing process (Figure S2). It is worth noting that even though the corresponding chemical dissimilarity between different domains is much less when compared to the chemical map of 1320 cm^–1^/1512 cm^–1^, the chemical contrast is still clearly shown, which implies that more hydroxyl groups would be concentrated in the graphene domain owing to the reaction of AEPIP and epoxy resin. Hence, the underlying mechanism of the selective localization of A-GNP in this multicomponent might be attributed to the electrostatic attractions between the A-GNP and AEPIP and/or the building block of epoxy-AEPIP generated during the polymerization. For further conformation, a similar blend lacking A-GNP was fabricated. As shown in Figure 4d, the contrast between the two phases is ambiguous and the extent of phase separation is too low to be detected by the optical images, which is one of the main obstacles impeding the investigation of the phase behavior of TS/TS blended systems.^21^ However, the chemical difference is clearly evident in the chemical maps shown in Figure 5e,f. The chemical distribution is consistent with the blend with A-GNP, indicating that the phase structure could be constructed without the introduction of A-GNP, while the incorporation of A-GNP highlights the phase structure constructed in this epoxy blend, enabling the separated phases to be observed without the necessity of an etching process, which is normally the method used to study the phase morphology of the phase-separated TS/TS blend.^21,28^ With the combination of the chemical mapping on both blends, it could be confirmed that the A-GNPs were selectively located in the domain constructed by the AEPIP-epoxy building blocks during the curing process.

In addition to accessing the chemical contrast of different microdomains, a second phase separation in the graphene domain has been detected by the chemical mapping with submicron resolution *via* O-PTIR spectroscopy (Figure 4b). It is found that the size of the second phase within the graphene microdomain is around 2 μm, which cannot be detected *via* the traditional FTIR spectroscopy and even infrared microspectroscopy. Generally, secondary phase separation would occur in a phase-separated structure in a thermoset blend and influence the performance. For instance, Masser et al.^29,30^ reported that the epoxy sample exhibits multiscale heterogeneity and higher ballistic impact resistance. Hence, to evaluate the effects of the concentration of AEPIP on the phase morphology evolutions (domain size and secondary phase separation), a specific epoxy blend A–BC (7:3) was fabricated. Figure 5a,b depicts the optical images of the A–BC (7:3) blend, which displays a phase behavior that is completely different from the A–BC (8:2) blend. It is apparent that the size of the secondary phase becomes much larger, making it easier to be observed. In the corresponding chemical map, it was found that even though the secondary phase displays the same blue color as the A–BC (8:2) blend, the larger circular microphase shows a lower concentration of AEPIP, unlike the A–BC (8:2) blend (Figure 4e). This implies that a converse phase structure would be constructed with the increase of AEPIP. More clear evidence was shown in the blend incorporating A-GNP (Figure 5d), where a larger epoxy-rich domain could be seen and isolated by the continuous graphene channel, which has been narrowed when compared to the A–BC (8:2) blend (Figure 1d).

**Figure 6:**
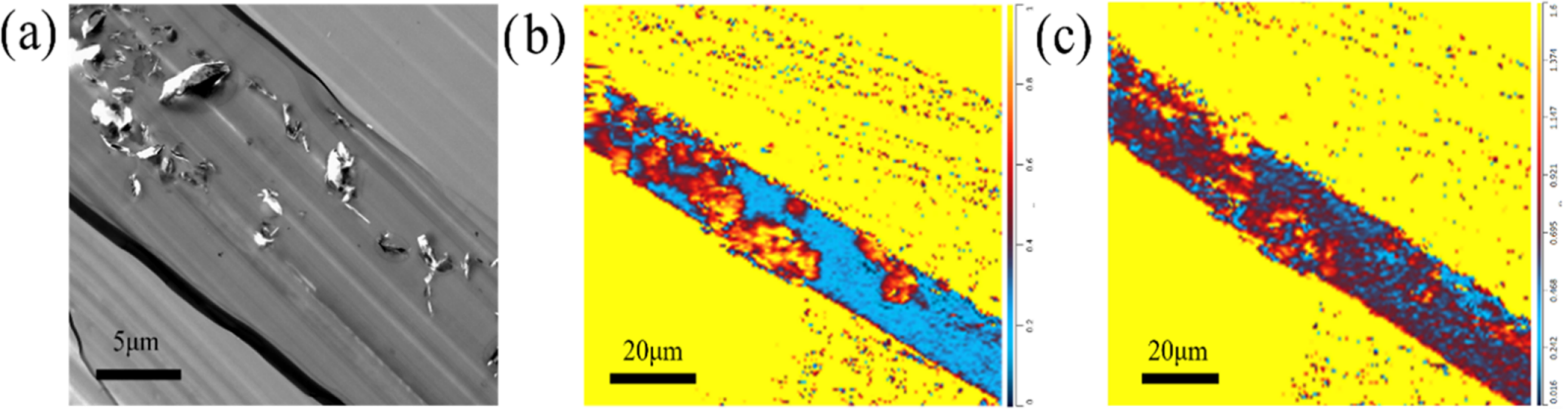
(a) TEM image and chemical map of (b) 1320 cm^–1^/1512 cm^–1^ and (c) 1030 cm^–1^/1512 cm^–1^ of the A–BC (7:3) blend with A-GNP.

To further explore the through-thickness phase morphology and related chemical distribution, TEM images and O-PTIR measurement were conducted on the cross section of the films cut *via* microtome. As shown in Figure 6a, it is evident that the graphene nanosheets undergo aggregation to form a column through the thickness (Figure 6a). Highlighting by the chemical map (Figure 6b,c), it could be confirmed that the graphene domain is not a big agglomeration of nanosheets but constructed both with graphene sheets and second phase-separated epoxy nanomicrodomains, simultaneously. Consequently, the composition of each domain was identified owing to this high-resolution O-PTIR mapping, providing critical indications for investigating the mechanism of selective localization in phase-separated structures in thermoset blends.

So far, in addition to the morphological analysis, such as AFM, SEM, TEM, and optical microscopy, which were the imaging techniques being relied on for decades for studying the phase behavior of TS blends, the O-PTIR technique reported here offers a novel and powerful approach for having a better understanding of the phase behavior of complex thermoset systems reinforced by nanofillers, highlighting the importance of accessing the chemical information to explain the phase structure and properties *via* O-PTIR spectroscopy. Furthermore, identifying the composition of the different micronanodomains for investigating the underlying mechanism provides a probability to design the materials with desired properties in a controllable manner,^21^ thus unlocking the potential application of these materials. However, due to the complexity of thermoset systems, for example, the mechanism of the selective localization of A-GNP in AEPIP-rich could not be confirmed yet. Therefore, work is continuing to figure out the underlying mechanism in the multicomponent thermoset blend for having a comprehensive understanding of this selective localization phenomenon.

## Conclusions

We have demonstrated for the first time the possibility of using O-PTIR spectroscopy to assist the investigation of the phase separation mechanism of TS blend with the selective localization of nanofillers. In contrast with the imaging (TEM, SEM, *etc*.) and thermal (DSC, DMA, *etc*.) characterization techniques, phase behavior was investigated by identifying the chemical distribution of components of the complex TS systems on the submicron scale using O-PTIR spectroscopy. Using this approach, compositions of different domains within the phase-separated structure produced from a simply modified commercial epoxy blend have been determined. The submicron resolution of this technique enables the detection of the chemical differences in the induced secondary phase separation within the graphene domains. However, the plausible mechanism of selectively locating A-GNP in the phase structure of the multicomponent blend could not be confirmed due to the complexity of thermoset systems and further exploration is necessary for a comprehensive understanding. Although it was not possible to determine the underlying phase separation mechanism directly through chemical mapping, this finding may provide new insight into the chemical distribution-phase structure relationship in TS blends, which normally presents as an important clue for investigating the mechanism. Furthermore, gaining an understanding of selectively locating nanofillers in the specific microdomain may help guide the design and production of these commercially important materials. Hence, the chemical analysis reported here affords a practical solution to the application of O-PTIR spectroscopy for assisting the investigation of the phase behavior of chemically complex systems at submicron resolution.

## Supporting Information Available

FTIR spectra of A-GNPs (Figure S1) and chemical reactions between epoxy and amine monomers (Figure S2) (PDF)
