# Supplementary material for: Analysis of the Chemical Distribution of Self-Assembled Microdomains with the Selective Localization of Amine-Functionalized Graphene Nanoplatelets by Optical Photothermal Infrared Microspectroscopy
Source: Anal Chem. 2022 Aug 16;94(34):11848–55. doi: 10.1021/acs.analchem.2c02306 (PMC9434550; doi:10.1021/acs.analchem.2c02306)
Supplement: Supplementary file 1 — ac2c02306_si_001.pdf [file ac2c02306_si_001.pdf]

Supporting information

Analysis of the Chemical Distribution of Self-assembled Microdomains with Selective Localization of Amine-functionalized Graphene Nanoplatelets by Optical Photothermal Infrared Microspectroscopy

Suihua He[1], Pascaline Bouzy [2], Nicholas Stone[2], Carwyn Ward[1], Ian Hamerton[1, *]

[1]Bristol Composites Institute, Department of Aerospace Engineering, School of Civil, Aerospace, and Mechanical, Engineering, University of Bristol, Queen's Building, University Walk, Bristol BS8 1TR, UK

[2]Physics and Astronomy, College of Engineering, Mathematics and Physical Sciences, University of Exeter, Exeter EX4 4QL, UK

***Corresponding author: ian.hamerton@bristol.ac.uk**

Contents of Supporting information

Figure S1. FTIR spectrum of amine-functionalized graphene nanoplatelets. S-2

Figure S2. Chemical reactions between epoxy and amine monomers. (a) Primary amine reaction, (b) Secondary amine reaction...S-3

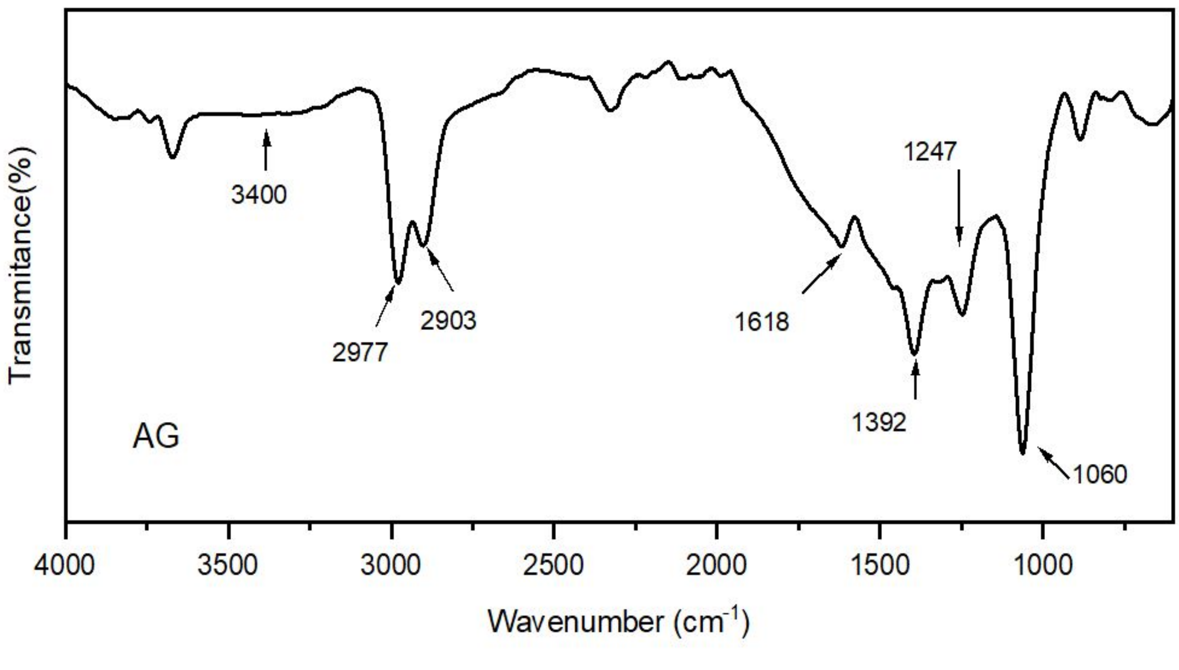

Figure S1. FTIR spectrum of amine-functionalized graphene nanoplatelets.

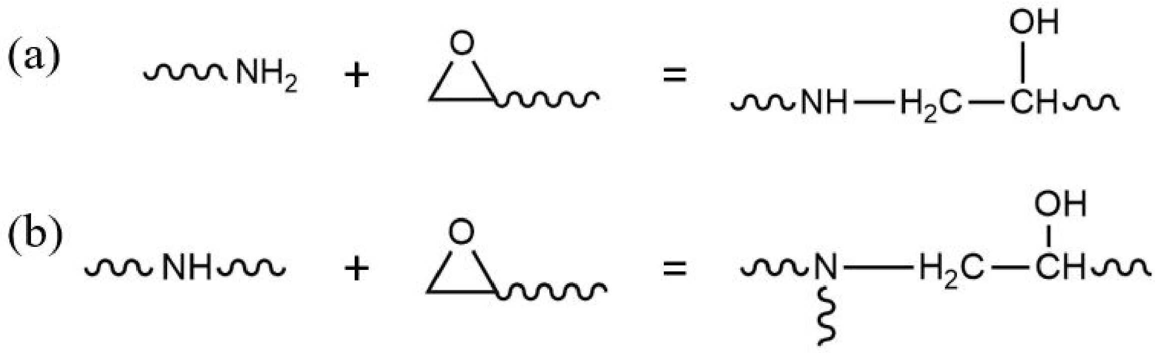

Figure S2. Chemical reactions between epoxy and amine monomers. (a) Primary amine reaction, (b) Secondary amine reaction.
